# Dynamics of Cognitive Function in Patients with Heart Failure Following Transcatheter Mitral Valve Repair

**DOI:** 10.3390/jcm11143990

**Published:** 2022-07-09

**Authors:** Muhammed Gerçek, Anca A. Irimie, Mustafa Gerçek, Henrik Fox, Vera Fortmeier, Tanja K. Rudolph, Volker Rudolph, Kai P. Friedrichs

**Affiliations:** 1 Clinic for General and Interventional Cardiology/Angiology, Herz- und Diabeteszentrum NRW, Ruhr-Universität Bochum, 32545 Bad Oeynhausen, Germany; vfortmeier@hdz-nrw.de (V.F.); trudolph@hdz-nrw.de (T.K.R.); vrudolph@hdz-nrw.de (V.R.); kpfriedrichs@hdz-nrw.de (K.P.F.); 2 Clinic for Neurology, Klinikum Herford, 32049 Herford, Germany; airimie@hdz-nrw.de; 3 Clinic for Cardiovascular Surgery, Herzzentrum Duisburg, 47137 Duisburg, Germany; mustafa.gercek@evkln.de; 4 Clinic for Thoracic and Cardiovascular Surgery, Herz- und Diabeteszentrum NRW, Ruhr-Universität Bochum, 32545 Bad Oeynhausen, Germany; hfox@hdz-nrw.de; 5 Heart Failure Department, Herz- und Diabeteszentrum NRW, Ruhr-Universität Bochum, 32545 Bad Oeynhausen, Germany

**Keywords:** transcatheter edge-to-edge repair, mitral regurgitation, cognitive function, Montreal Cognitive Assessment Test

## Abstract

Aims: Interventional transcatheter edge-to-edge mitral valve repair (TMVR) is an established treatment option for patients with severe mitral regurgitation (MR) and high operative risk. Cognitive impairment is one of the most common conditions among often extensive comorbidities in these patients. The specific patterns of cognitive decline and particularly the effect of TMVR are not well described. Thus, this study aimed to investigate into the impact of TMVR on cognitive impairment, exercise capacity, and quality of life. Methods: Cognitive function (executive, naming, memory, attention, language, abstraction, and orientation) was assessed with the standardized Montreal Cognitive Assessment test (MoCA; range between 0 and 30 points) before and 3 months after TMVR in 72 consecutive patients alongside echocardiographic examination and assessment of exercise capacity (six-minute walk test) as well as quality-of-life questionnaires (Minnesota living with heart failure questionnaire, MLHF-Q). Results: Patients’ median age was 81 [76.0; 84.5] years, 39.7% were female with a median EuroScore II of 4.4% [2.9; 7.7]. The assessment of cognitive function showed a significant improvement of the cumulative MoCA-Test result (from 22.0 [19.0; 24.5] to 24 [22.0; 26.0]; *p* < 0.001) with significant changes in the subcategories executive (*p* < 0.001), attention (*p* < 0.001), abstraction (*p* < 0.001), and memory (*p* < 0.001). In addition, quality of life (from 47.5 [25.0; 69.3] to 24.0 [12.0; 40.0]; *p* < 0.001) and exercise capacity (from 220.0 m [160.0; 320.0] to 280.0 m [200.0; 380.0]; *p* = 0.003) increased significantly 3 months after the TMVR procedure. Conclusions: TMVR leads to a significant improvement of cognitive function, exercise capacity, and quality of life in patients with chronic heart failure in 3 months follow up and again highlights the benefit of the evermore established TMVR procedure for patients with high operative risk.

## 1. Introduction

Mitral regurgitation (MR) is associated with increased mortality in heart failure and reduces quality of life dramatically [1]. In patients with higher surgical risk, transcatheter edge-to-edge mitral valve repair (TMVR) has been well established and has received a class IIa recommendation for primary mitral regurgitation (PMR) as well as secondary mitral regurgitation (SMR) [2,3]. Regarding SMR, two randomized trials revealed that TMVR using MitraClip devices (Abbott, Chicago, IL, USA) is safe and effective in reducing MR [4,5]. Even though these two studies’ results differed in terms of prognostic effectiveness, several post-hoc analyses revealed the beneficial effects of TMVR using the MitraClip device particularly with regard to clinical improvement such as quality of life and exercise capacity [6].

Meanwhile, a second safe and effective TMVR device, PASCAL (Edwards, Irvine, CA, USA), was introduced that generated huge momentum in the field of transcatheter mitral valve intervention [7]. Henceforth, independent leaflet capture with PASCAL- and newer generations of the MitraClip devices were possible, and thus enabled the treatment of more complex mitral valve anatomies [8]. 

The association between heart failure as well as MR and burdensome impairment of cognitive function is known [9]. The brain, being one of the human body’s most demanding organs, requires up to 20% of total oxygen and glucose supply [10]. Maintaining an effective cerebral perfusion is paramount to guarantee a stable neuronal homeostasis to preserve the brain´s structural and functional integrity [10]. Cerebral perfusion can still be maintained to a certain extent in spite of disturbed hemodynamics or reduced pressure [9]. However, prolonged exposure to impaired hemodynamics, which are present in the context of heart failure and MR, can lead to reduced cerebral autoregulation with decreased cerebral blood flow resulting in neuronal injury and cognitive alteration [11]. 

Considering the beneficial effects of TMVR on exercises capacity and quality of life, we hypothesize that TMVR may also have a favorable effect on cognitive function. 

## 2. Methods

### 2.1. Study Population 

**Figure 1:**
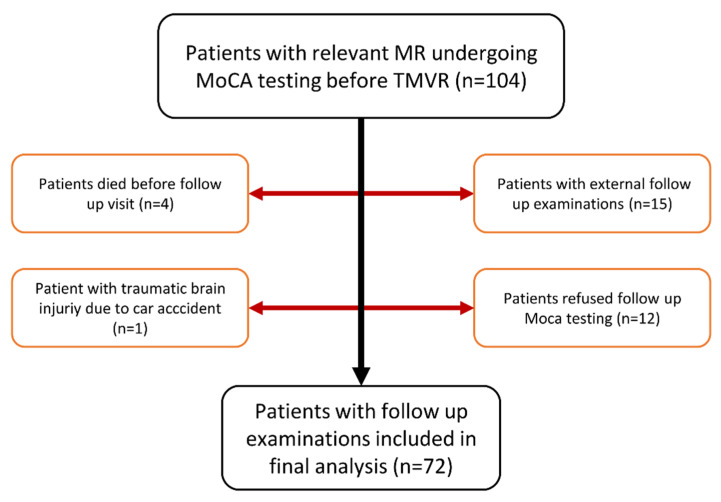
Flow chart for patient recruitment. MR = Mitral regurgitation; TMVR = Transcatheter mitral valve repair; MoCA = Montreal cognitive assessment.

In this prospective single center study, 104 patients who underwent TMVR for relevant MR and symptomatic heart failure (NYHA ≥ II) received an assessment of cognitive function with the Montreal cognitive assessment (MoCA) test at baseline before intervention. TMVR was performed using the PASCAL- (Edwards Lifesciences, Irvine, CA, USA) or the MitraClip-device (Abbott, Chicago, IL, USA). Four patients died before the follow up visit, 1 patient was excluded because of a traumatic brain injury due to a car accident, 15 patients received follow up examinations at external institutions, and 12 patients refused follow up examinations of their cognitive function. In total, 72 patients were included in the final analysis (Figure 1).

Exams were performed before and 3 months after the procedure between January 2019 and December 2020. Exercise capacity was assessed using the 6 minutes walking test. Clinical assessments were performed using the New York Heart Association classification and the Minnesota living with heart failure questionnaire (MLHFQ), along with laboratory evaluation of amino terminal pro-brain natriuretic peptide (NTpro-BNP) as a cardiac marker for congestive heart failure.

All study participants underwent standard transthoracic echocardiography. The echo studies were performed by highly qualified medical staff and analyzed by the same echocardiographer with long-time experience. The analyses and grading of the mitral regurgitation were performed following the recommendations of the American and European Societies of Echocardiography and according to previously published guidelines [12,13]. Data Collection was approved by the local Ethics Committee of the Ruhr University Bochum (Germany) and carried out in accordance with the Declaration of Helsinki. Written informed consent was obtained from every patient (2018-383) and all data were included in a database which is registered at www.clinicaltrials.gov (accessed on 4 June 2022) (NCT03813511).

### 2.2. Assessment of the Cognitive Function Using the Montreal Cognitive Assessment Test

The Montreal Cognitive Assessment (MoCA) test is a screening test to detect cognitive impairment. It assesses different cognitive domains such as attention and concentration, executive function, memory, language, visuoconstructional skills, conceptual thinking, calculation, and orientation. The test and its instructions are available online (www.mocatest.org (accessed on 4 June 2022)). The MoCA test has been shown to precisely detect mild cognitive impairment in patients over 60 years [14,15]. It is ranked out of a maximum of 30 points, while a score of 26 or above is considered normal. In our setting, the test was performed by the same experienced neurologist at baseline and follow-up visits. The neurologist was blinded with regard to the outcome of the intervention in terms of MR reduction or any other clinical information to minimize further investigator-related bias. To mitigate training-dependent improvement, different MoCA test versions were used at baseline and follow-up.

### 2.3. Statistical Analysis 

Statistical analysis was performed using the SPSS-Software (Version 22, IBM Corporation, Armonk, NY, USA). Continuous variables are reported as mean ± standard deviation (SD) when normally distributed, otherwise as median and interquartile range (IQR). Categorical variables are presented as frequencies and percentages. Student’s *t*-test for unpaired and paired parametric samples or their analogues for nonparametric samples (Mann-Whitney-U-test and Wilcoxon-signed rank) or the chi-square test were performed for group comparisons, when appropriate. A *p*-value < 0.05 was considered to indicate statistical significance.

## 3. Results

### 3.1. Baseline Characteristics

Patients’ median age was 81 [76.0; 84.5] years; 39.7% were female. Surgical risk was on average moderate with an EuroScore II of 4.4% [2.9; 7.7] and an STS-Score of 2.5% [1.5; 3.9]. Detailed characteristics are provided in Table 1. PMR was present in 42.5% of the cases. The left atrium was on average severely dilated (LA volume index 66.0 mL/m^2^ [51.5; 82.5]) and the left ventricle moderately dilated (LV end-diastolic diameter (57.0 mm [50.0; 61.0]). Additionally, the systolic pulmonary artery pressure was elevated (43.5 ± 16.9 mmHg). Echocardiographic parameters are listed in Table 2.

### 3.2. Procedural Outcome

TMVR using PASCAL was performed in 38 cases (52.8%), while the MitraClip was employed in 34 (47.2%) cases. Technical success could be achieved in all cases. MR reduction ≥2 grades could be achieved in 64 (88.8%) cases. Procedure time was 89.0 min [65.3; 113.0] with a fluoroscopy time of 7.8 min [5.8; 12.4]. On average, 1.0 device [1.0; 2.0] was implanted. Two patients suffered from major bleeding and required blood transfusion. No other interventions were needed. The postprocedural transmitral gradient was acceptable (3.0 mmHg [2.3; 4.0]) and remained stable at follow up (*p* = 0.49). All Patients were discharged in good clinical condition on the second [2.0; 3.0] postinterventional day. 

### 3.3. Echocardiography Results

Follow up echocardiography was performed on average 106.5 ± 59.7 days postprocedurally.

**Figure 2:**
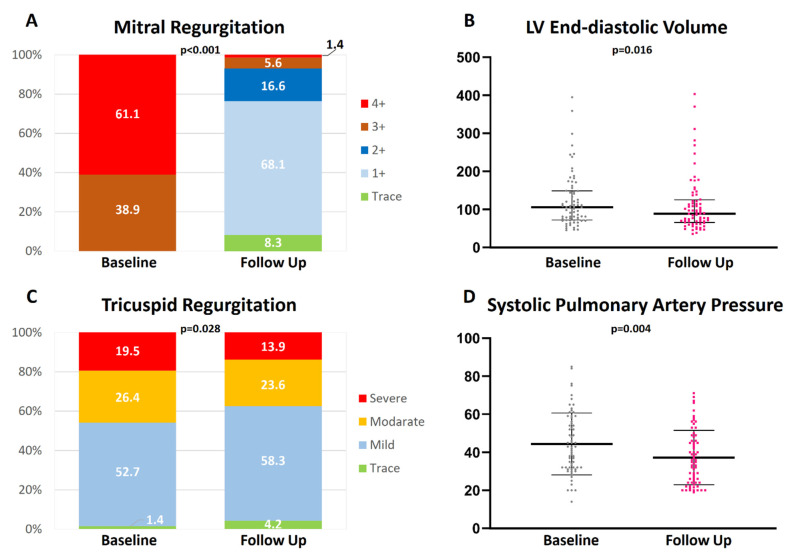
Echocardiographic examinations in patients undergoing transcatheter edge-to-edge mitral valve repair (TMVR). TMVR results in sustained reduction of mitral regurgitation (**A**), slight left ventricular (LV) remodeling (**B**), and reduction of tricuspid regurgitation (**C**) as well as pulmonary artery pressure (**D**).

Sustained MR ≤ 1+ was present in 76.4% of the patients (Figure 2). Left ventricular dimensions showed a significant reduction (LV end-diastolic diameter: from 57.0 mm [50.0; 61.0] to 53.0 mm [46.5; 60.0]; *p* = 0.009 and LV end-diastolic volume from 104.0 mL [72.3; 148.8] to 88.0 mL [66.5; 124.0]; *p* = 0.016), whereas left atrial volumes did not differ significantly (*p* = 0.12).

Baseline MR grade was significantly higher in patients with PMR (PMR > 3 + 92.1% vs. SMR > 3 + 36.4%; *p* < 0.001), which is why MR reduction > 2 grades was significantly higher in PMR patients (MR reduction in PMR 3.0 [2.0; 3.0] vs. MR reduction in SMR 2.0 [2.0; 3.0]; *p* = 0.023). However, MR ≥ 1+ at the end of the procedure was comparable between PMR and SMR patients (72.7% vs. 79.5%; *p* = 0.51).

At follow up, tricuspid regurgitation (TR) significantly improved from TR ≥ II in 42.4% of the cases to 27.7%. Additionally, systolic pulmonary artery pressure decreased significantly (from 43.5 ± 16.9 mmHg to 36.6 ± 15.7 mmHg; *p* = 0.004). Echocardiographic parameters are listed in detail in Table 2. Patients with PMR showed a significantly higher improvement of quality of life than patients with SMR (22.1 ± 22.8 vs. 10.2 ± 18.3; *p* = 0.013). Otherwise, no significant difference was detected (Supplementary Table S1). There was no significant difference in echocardiographic parameters regarding the chosen TMVR device (PASCAL vs. MitraClip) (Supplementary Table S2). 

#### Cognitive Function and Clinical Parameters Improved after TMVR

Fifty percent of the patients scored below the MoCA test cut off value (−1 to −2 standard deviation) [16]. Patients reached an average total score of 22.0 [19.0; 24.5] at baseline. Cognitive dimension such as naming, language, and orientation were the most preserved cognitive capacities and did not show a significant change at follow up. The worst results at baseline were observed in executive function 3.0 [2.0; 4.0] and memory 2.0 [1.0; 3.0]. 

**Figure 3:**
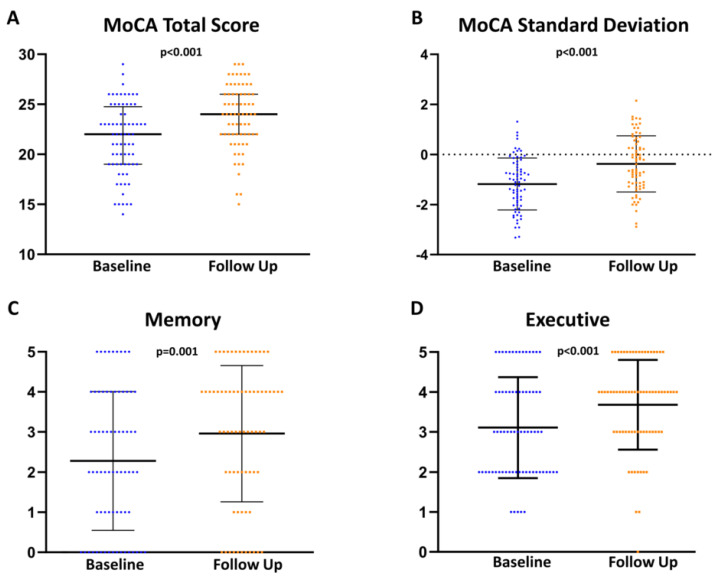
Transcatheter edge-to-edge mitral valve repair (TMVR) results in significant improvement in Montreal cognitive assessment (MoCA) testing. The total Score in MoCA (**A**) and the adjusted score for age and education (**B**) improved significantly after TMVR. Memory (**C**) and executive function (**D**) showed the highest improvement.

At follow up, patients reached a significantly higher total score in MoCA testing, particularly in the cognitive dimensions of executive function and memory (Figure 3). Detailed MoCA results are provided in Table 3. With regard to the etiology of the mitral regurgitation, no significant differences in the MoCa results could be determined (Supplementary Table S3).

**Figure 4:**
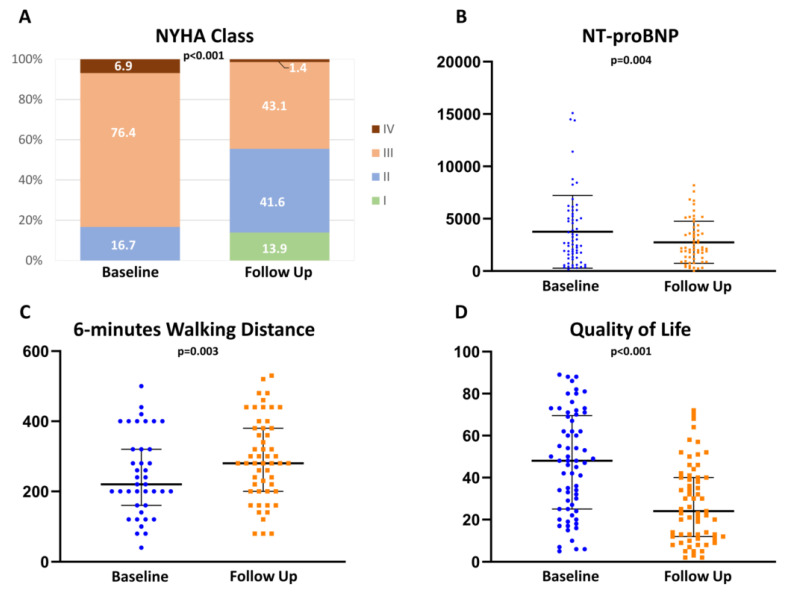
Transcatheter edge-to-edge mitral valve repair (TMVR) results in significant clinical improvement. New York Heart Association (NYHA) class (**A**), amino terminal pro-brain natriuretic peptide (NT-proBNP) (**B**), 6-min walking distance (**C**), and quality of life (**D**) presented a remarkable improvement at follow up.

Furthermore, patients reported a significant improvement in NYHA class (from 83.3% ≥ III to 55.5% ≤ II; *p* < 0.001) as well as in quality of life (from 47.5 [25; 69.3] to 24.0 [12.0; 40.0]; *p* < 0.001) and achieved a significantly increased walking distance (from 220.0 m [160.0; 320.0] to 280.0 m [200.0; 380.0] m; *p* = 0.003) in the 6 minutes walking test (Figure 4). 

## 4. Discussion

This is the first prospective study on mitral regurgitation that assesses cognitive function with the MoCA test before and after TMVR. The main findings are that in our cohort of MR patients a high percentage of at least mild cognitive impairment at baseline could be detected (I), and TMVR resulted in an increased MoCA score with the strongest improvement in executive function and memory (II). Alongside improvements in exercise capacity, TMVR has beneficial effects on several aspects such as cognitive function, which might contribute to increased quality of life (III). 

In elderly patients with several comorbidities, a gap in-between normal cognitive aging and dementia can be observed [17]. This is generally referred to as cognitive impairment and is often observed in patients with heart failure since the brain’s high metabolic demand cannot be fully met in these patients [18]. The chronically lower blood flow to the brain could enable a neuronal metabolic energy crisis and lead to oxidative stress that promotes increased tau phosphorylation, mitochondrial dysfunction, and astrocyte dysregulation, which is known to lead to neurodegeneration [19].

Thus, identifying mild cognitive impairment in symptomatic MR patients with high surgical risk due to multiple comorbidities seems intuitive. Hemodynamic abnormalities are a hallmark of heart failure and valve insufficiencies, which can reduce cardiac output [9]. The imbalance of the autonomous nervous system featuring a prevalence of the sympathetic tone over vagal activity is an important underlying neuro-hormonal mechanism in heart failure pathogenesis. This, at least partly, contributes to affecting normal cognitive function in these patients [20]. While explicitly acknowledging that cognitive impairment may be the result of multifactorial pathophysiology, especially in elderly patients with a natural course of brain aging, heart failure and reduced cardiac output are well associated with impaired neurocognitive functions. This is well known and accordingly the term “cardiogenic dementia” has been coined [18,21]. Reduced cardiac output can lead to inconsistencies in cerebral perfusion and thus to misguided cerebral metabolism resulting in an increased vulnerability for cerebral hypoxia [22]. The hippocampus and posterior cingulate cortex are some of the most affected regions in cerebral hypoperfusion and are known to play a key role in cognitive function such as memory [23]. Another subtle interaction was observed between baseline cardiac output and APOE-ε4 carrier status on longitudinal information processing speed and episodic memory performances [24].

Previous studies suggested an association between MR in heart failure patients and cognitive dysfunction, but have so far solely focused on executive function and memory [25]. Using the MoCA test, we gained a more holistic overview of the cognitive functions and can confirm cognitive impairments not only in executive function and memory but also in abstraction and attention. 

TMVR procedures were performed using both the MitraClip- and PASCAL-system. Both procedures were safe and effective in achieving a high percentage of MR reduction (88.9%). 

At follow-up, tricuspid regurgitation and systolic pulmonary artery pressure decreased alongside left ventricular end-diastolic volume, indicating improved cardiac output (Figure 2). 

Accordingly, clinical parameters such as NYHA class, 6 minutes walking distance, and quality of life improved. In MoCA testing, executive function and memory showed the highest improvements 3 months after TMVR. Thus, our study is in line with the literature and reinforces the assumption that cognitive function improves due to a re-established effective hemodynamic situation [26].

Terhoeven et al., also reported that patients who underwent TMVR for SMR showed significant improvements in memory and executive function in particular in patients with the worst baseline values for cognitive dimensions [26]. Our study clearly underlines the positive effects of TMVR in primary as well as secondary SMR in terms of cognitive function and exercise capacity. As a result, restoring cognitive skills and exercise tolerance may be of tremendous benefit in facing daily life challenges and maintaining an adequate self-care and quality of life. 

Several limitations apply to our study. A recognition/training effect in MoCA testing is possible. Participants can achieve a higher score due to the recognition of the task and question, which might result in an overestimation of improvement in the MoCA score during the follow up examinations [27]. To mitigate this possibility, the MoCA test was performed by a highly experienced neurologist (>10 years of experience) and by using two different versions of the test during the initial and follow up examinations. Additionally, the investigator was blinded to the procedural outcome of the patients, to avoid an investigator-related bias, and thereby minimizing the risk of over- or underestimating patient performance in MoCA testing. Unfortunately, the number of patients excluded from the final analysis is high, which is a significant limitation of our study. Fifteen patients received follow-up examinations in external facilities because the distance to our center, to which patients were referred from all over the country, was too far, so they preferred local doctors for follow-up visits. Some patients declined follow-up tests of their cognitive function because of unsatisfactory results at the initial visit, which they described as too stressful for them. However, the overall MoCA results at baseline were not significantly different from those of the final study cohort (22.0 [19.0; 24.5] vs. 21.0 [20.0; 24.0]; *p* = 0.71), so we would expect the same effect of TMVR on their cognitive function. Furthermore, our study is of descriptive nature and thus not designed to explain the phenomena it observes and can only generate hypotheses. 

## 5. Conclusions

Cognitive impairment was identified in 50% of the patients with relevant mitral regurgitation undergoing TMVR. However, TMVR results in an increased MoCA Score with the strongest improvement in executive function and memory, improved cognitive function, alongside increased exercise capacity and quality of life. Restoring cognitive skills and exercise tolerance may be of tremendous benefit in facing daily life challenges and maintaining adequate self-care.

## Figures and Tables

**Table 1:** Baseline Characteristics.

Characteristics	
Age	81.0 [76.0; 84.5]
Female	39.7% (29)
Body mass index kg/m^2^	26.2 [23.0; 29.2]
EuroScore II (%)	4.4 [2.9; 7.7]
STS Score for mitral valve repair	2.5 [1.5; 3.9]
Atrial fibrillation	63.0% (46)
Diabetes mellitus	12.3% (9)
Chronic obstructive pulmonary disease	16.7% (12)
Coronary artery disease	46.6% (34)
History of myocardial infarction	13.7% (10)
History of cardiac surgery	24.7% (18)
Stroke	27.4% (20)
Dialysis	2.7% (2)
NTpro-BNP [pg/mL] *	2680.0 [1520.0; 5292.5]

* Dialysis patients were excluded from the analysis. NTpro-BNP: amino terminal pro-brain natriuretic peptide.

**Table 2:** Comparison of baseline and follow up echocardiographic imaging parameters.

Imaging Parameters	Baseline	Follow Up	*p*-Value
LV ejection fraction [%]	51.9 ± 15.2	49.4 ± 14.0	0.51
LV end-diastolic diameter [mm]	57.0 [50.0; 61.0]	53.0 [46.5; 60.0]	0.009 *
LV end-diastolic volume [mL]	104.0 [72.3; 148.8]	88.0 [66.5; 124.0]	0.016 *
LV end-systolic diameter [mm]	39.0 [34.0; 50.0]	37.0 [32.0; 48.8]	0.82
LV end-systolic volume [mL]	46.0 [32.0; 79.8]	39.0 [28.5; 69.0]	0.40
LA volume [mL]	121.0 [92.5; 153.0]	108.0 [78.8; 140.3]	0.20
LA volume index [mL/m^2^]	66.0 [51.5; 82.5]	59.0 [45.0; 77.0]	0.12
Mitral regurgitation etiology			
Primary Secondary Mixed	431% (31) 54.2% (39) 2.8% (2)	∅	∅
Mitral regurgitation grade			
Trace Mild Mild-to-Moderate Moderate-to-Severe Severe	∅ ∅ ∅ 38.9% (28) 61.1% (44)	8.3% (6) 68.1% (49) 16.6% (12) 5.6% (4) 1.4% (1)	<0.001 ^+^
MR vena contracta width [mm]	8.0 [6.0; 11.0]	3.0 [2.0; 4.0]	<0.001 *
MR EROA [cm^2^]	0.4 [0.2; 0.7]	0.1 [0.1; 0.1]	<0.001 *
MR regurgitant volume [mL]	56.0 [35.5; 89.5]	13.0 [7.0; 20.0]	<0.001 *
Mean transmitral gradient [mmHg]	2.0 [1.0; 3.0]	3.0 [2.0; 4.0]	<0.001 *
Degree of tricuspid regurgitation			
Trace Mild Moderate Severe	1.4% (1) 52.7% (38) 26.4% (19) 19.5% (14)	4.2% (3) 58.3% (40) 23.6% (17) 13.9% (10)	0.003 ^+^
Systolic pulmonary artery pressure [mmHg]	43.5 ± 16.9	36.6 ± 15.7	0.004 ^#^

EROA = effective regurgitation orifice area, LA = left atrial, LV = left ventricular, MR = mitral regurgitation. ^#^ = paired *t*-test; * = Wilcoxon signed-rank test; ^+^ = χ^2^-Test.

**Table 3:** Comparison of baseline and follow up results in the MoCA test.

Parameter	Baseline	Follow Up	*p*-Value
MoCA result	22.0 [19.0; 24.5]	24.0 [22.0; 26.0]	<0.001 *
MoCA standard deviation results	−1.2 ± 1.0	−0.4 ± 1.1	<0.001 ^#^
Executive Function	3.0 [2.0; 4.0]	4.0 [3.0; 4.5]	<0.001 *
Naming	3.0 [3.0; 3.0]	3.0 [3.0; 3.0]	0.52
Memory	2.0 [1.0; 3.0]	3.0 [2.0;4.0]	<0.001 *
Attention	5.0 [4.0; 6.0]	6.0 [5.0; 6.0]	<0.001 *
Language	2.0 [1.0; 2.0]	2.0 [1.0; 2.0]	0.33
Abstraction	1.0 [1.0; 2.0]	2 [2.0; 2.0]	<0.001 *
Orientation	6.0 [6.0; 6.0]	6.0 [6.0; 6.0]	0.39
6-min-walking distance	220.0 [160.0; 320.0]	280.0 [200.0; 380.0]	0.003 *
Quality of Life (MLHFQ)	47.5 [25; 69.3]	24.0 [12.0; 40.0]	<0.001 *
NTproBNP	2680.0 [1520.0; 5292.5]	2150.0 [1232.5; 3847.5]	0.004 *

MoCA = Montreal Cognitive assessment; MLHFQ= Minnesota living with heart failures questionnaire. NTpro-BNP = amino terminal pro-brain natriuretic peptide. ^#^ = *t*-test; * = Wilcoxon signed-rank test.

## Data Availability

The data underlying this article will be shared upon reasonable request to the corresponding author.

## Supplementary Materials

The following supporting information can be downloaded at: https://www.mdpi.com/article/10.3390/jcm11143990/s1, Table S1: Comparison of echocardiographic and clinical delta values in PASCAL and MitraClip; Table S2: Comparison of echocardiographic and clinical delta values in primary and secondary mitral regurgitation; Table S3: Comparison of delta values in the MoCA test in primary and secondary mitral regurgitation.
