# Supplementary material for: Dynamics of Cognitive Function in Patients with Heart Failure Following Transcatheter Mitral Valve Repair
Source: J Clin Med. 2022 Jul 9;11(14):3990. doi: 10.3390/jcm11143990 (PMC9317412; doi:10.3390/jcm11143990)
Supplement: Supplementary file 1 [file jcm-11-03990-s001.zip › jcm-1781577-supplementary.pdf]

Table S1. Comparison of echocardiographic and clinical delta values in PASCAL and MitraClip.

Parameter<br>Δ (Baseline – Follow Up)	PASCAL<br>(N=38)	MitraClip<br>(N=34)	p-value
**LV ejection fraction [%]**	1.4 ± 10.9	3.5 ± 10.0	0.40
**LV end-diastolic diameter [mm]**	1.5 [2.0; 6.0]	3.0 [2.0; 8.0]	0.45
**LV end-diastolic volume [ml]**	16.5 [4.0; 29.3]	9.5 [9.5; 32.5]	0.94
**LV end-systolic diameter [mm]**	0.3 ± 10.5	1.3 ± 15.1	0.76
**LV end-systolic volume [ml]**	4.0 [3.0; 16.0]	3.0 [8.3; 17.0]	0.88
**LA volume [ml]**	-2.6 ± 41.4	-11.2 ± 44.6	0.40
**LA volume index [ml/m²]**	-1.2 ± 22.3	-7.6 ± 23.0	0.24
**Reduction of Mitral regurgitation**	3 [2.0; 3.0]	2.0 [2.0; 3.0]	0.14
**MR vena contracta width [mm]**	6.0 [3.3; 8.8]	3.5 [2.0; 6.8]	0.06
**MR EROA [cm²]**	0.4 [0.2; 0.7]	0.2 [0.1; 0.5]	0.57
**MR regurgitant volume [ml]**	50.7 ± 41.4	36.9 ± 36.8	0.15
**Systolic pulmonary artery pressure [mmHg]**	3.8 ± 16.6	9.0 ± 16.6	0.22
**NTproBNP**	161.0 [0.0; 1267.5]	1.0 [-267.0; 1107.5]	0.92
**NYHA Class**	0.0 [0.0; 1.0]	1.0 [0.0; 1.0]	0.27
**6-min-walking distance**	30.0 [8.0; 60.0]	20.5 [5.5; 80.0]	.099
**Quality of life (MLHFQ)**	13.5 [4.8; 31.3]	7.5 [0.0; 20.3]	0.23

EROA= effective regurgitation orifice area, LA= left atrial, LV= left ventricular, MR= mitral regurgitation; NT-proBNP: amino terminal pro-brain natriuretic peptide

Table S2. Comparison of echocardiographic and clinical delta values in primary and secondary mitral regurgitation.

Parameter<br>Δ (Baseline – Follow Up)	Primary MR<br>(N=31)	Secondary MR<br>(N=39)	p-value
**LV ejection fraction [%]**	3.6 ± 9.1	1.1 ± 11.5	0.38
**LV end-diastolic diameter [mm]**	2.0 [-4.0; 6.0]	3.0 [-2.0; 5.0]	0.31
**LV end-diastolic volume [ml]**	17.0 [1.0; 52.0]	10.0 [-12.0; 28.0]	0.09
**LV end-systolic diameter [mm]**	1.6 ± 11.9	0.1 ± 13.9	0.76
**LV end-systolic volume [ml]**	4.0 [-3.0; 20.0]	3.5 [-8,5; 12.3]	0.48
**LA volume [ml]**	17.3 ± 43.6	0.8 ± 40.6	0.07
**LA volume index [ml/m²]**	9.3 ± 24.9	0.9 ± 20.1	0.07
**Reduction of mitral regurgitation**	3.0 [2.0; 3.0]	2.0 [2.0; 3.0]	**0.023***
**MR vena contracta width [mm]**	8.0 [6.0; 9.0]	3.0 [2.0; 5.0]	**<0.001***
**MR EROA [cm²]**	0.6 [0.5; 0.8]	0.2 [0.1; 0.2]	0.50
**MR regurgitant volume [ml]**	70.7 ± 41.3	24.9 ± 24.6	**<0.001#**
**Systolic pulmonary artery pressure [mmHg]**	10.5 ± 16.1	2.8 ± 16.9	0.09
**NTproBNP**	173.0 [0.0; 1240.0]	0.5 [-372.5; 1061.3]	0.76
**NYHA Class**	0.0 [0.0; 1.0]	0.0 [0.0; 1.0]	0.47
**6-min-walking distance**	40.0 [15.5; 74.0]	18.5 [3.8; 57.5]	0.06
**Quality of life (MLHFQ)**	14.0 [5.0; 32.0]	8.0 [0.0; 19.0]	**0.013***

EROA= effective regurgitation orifice area, LA= left atrial, LV= left ventricular, MR= mitral regurgitation; NT-proBNP: amino terminal pro-brain natriuretic peptide; # = unpaired t-test; * = Mann-Whitney-U-test

Table S3. Comparison of delta values in the MoCA test in primary and secondary mitral regurgitation.

Parameter<br>Δ (Follow Up-Baseline)	Primary MR<br>(N=31)	Secondary MR<br>(N=39)	p-value
**MoCA result**	2.0 [0.0; 4.0]	2.0 [1.0; 4.0]	0.33
**MoCA standard deviation results**	0.7 ± 0.8	0.8 ± 1.0	0.68
**Executive function**	1.0 [0.0; 1.0]	1.0 [0.0; 1.0]	0.42
**Naming**	0.0 [0.0; 0.0]	0.0 [0.0; 0.0]	0.80
**Memory**	1.0 [0.0; 1.0]	1.0 [0.0; 2.0]	0.11
**Attention**	0.0 [0.0; 1.0]	0.0 [0.0; 1.0]	0.89
**Language**	0.0 [0.0; 1.0]	0.0 [-1.0; 1.0]	0.11
**Abstraction**	0.0 [0.0; 1.0]	1.0 [0.0; 1.0]	0.21
**Orientation**	0.0 [0.0; 0.0]	0.0 [0.0; 0.0]	0.93

MR= mitral regurgitation; MoCA= Montreal Cognitive Assessment Test.
